# Evaluation of the Self-Healing Ability of Mortar Mixtures Containing Superabsorbent Polymers and Nanosilica

**DOI:** 10.3390/ma13020380

**Published:** 2020-01-14

**Authors:** Gerlinde Lefever, Didier Snoeck, Dimitrios G. Aggelis, Nele De Belie, Sandra Van Vlierberghe, Danny Van Hemelrijck

**Affiliations:** 1 Department Mechanics of Materials and Constructions, Vrije Universiteit Brussel (VUB), Pleinlaan 2, 1050 Brussels, Belgium; didier.snoeck@ugent.be (D.S.); Dimitrios.aggelis@vub.be (D.G.A.); Danny.Van.Hemelrijck@vub.be (D.V.H.); 2 Magnel-Vandepitte Laboratory for Building Materials and Structures, Department of Structural Engineering and Building Materials, Faculty of Engineering and Architecture, Ghent University, Tech Lane Ghent Science Park, Technologiepark Zwijnaarde 60, 9052 Ghent, Belgium; Nele.DeBelie@UGent.be; 3 Polymer Chemistry & Biomaterials Research Group, Centre of Macromolecular Chemistry, Ghent University, Krijgslaan 281 S4-Bis, 9000 Ghent, Belgium; sandra.vanvlierberghe@ugent.be

**Keywords:** self-healing concrete, water permeability, crack width measurement, hydrogels, nanosilica

## Abstract

Addition of superabsorbent polymers (SAPs) to cementitious mixtures promotes the self-healing ability of the material. When cracking occurs; SAPs present inside the crack will swell upon contact with water and subsequently release this water to stimulate the further hydration of unhydrated cement particles and the calcium carbonate crystallization. However; the inclusion of SAPs affects the mechanical performance of the cementitious material by the creation of macro-pores as water is retracted from the swollen SAP. To counteract the reduction in strength, part of the cement is replaced by nanosilica. In this research, different mixtures containing either SAPs or nanosilica and a combination of both were made. The samples were subjected to wet–dry cycles simulating external conditions, and the self-healing efficiency was evaluated by means of the evolution in crack width, by optical measurements, and a water permeability test. In samples containing SAPs, an immediate sealing effect was observed and visual crack closure was noticed. The smaller influence on the mechanical properties and the good healing characteristics in mixtures containing both nanosilica and SAPs are promising as a future material for use in building applications.

## 1. Introduction

Concrete is known to be highly sensitive to cracking, due to its low tensile strength, which increases the possibility of aggressive substances entering the concrete element and causing damage to both the cementitious matrix and the reinforcement material. For this reason, the repair of concrete structures is of utmost importance and comprises around 50% of the annual construction budget in Europe [1]. These high costs, combined with the fact that most repairs are only temporary solutions, lead to the introduction of self-healing concrete. This type of concrete is able to inherently repair cracks, so that no external maintenance is needed and additional costs are limited.

Several techniques for self-healing in cementitious media have been investigated and subdivided into autogenous and autonomous healing. In the case of autonomous healing, a healing agent is encapsulated inside the concrete material to repair the cracks [2,3]. Autogenous healing concerns all methods that use the self-healing capability of the cementitious matrix itself. Healing is, in this case, obtained by means of further hydration of cement particles or recrystallization and carbonation of Ca(OH)_2_ [4]. A method that is currently of great interest is the use of superabsorbent polymers (SAPs) as internal water reservoirs, promoting self-healing of cementitious mixtures [5,6,7]. When cracking occurs and water or moisture enters the cracks, the SAPs are able to absorb this water and retain it for a certain period of time. Not only will the swelling of the SAPs block the entrance to the cracks (self-sealing), they will also release the absorbed water later on to allow for further hydration of cement particles inside the cracks (self-healing). Moreover, repeated healing can be obtained as the SAPs are able to swell and shrink multiple times during a structure’s service life [8]. Even more so, the autogenous healing is promoted up to a minimum concrete age of 8 years [9]. One of the techniques that is often used to confirm self-sealing and self-healing is a water permeability test, during which the water flow through the crack opening is measured. Various researchers have studied the influence of SAPs on this water flow and found that SAPs were effective for both immediate self-sealing and for self-healing after application of wet–dry healing cycles [7,10,11].

Besides the concerns regarding cracking during concrete’s service life, there is also the possibility of matrix cracking before the structure is subjected to external loads. In the case of a low water-to-cement ratio, the increase in cracking due to early-age shrinkage should also be taken into account. As Powers’ hydration model described [12,13], a water-to-cement ratio of 0.42 is needed to reach full hydration of cement particles. Whenever the water content is below this value, self-desiccation takes place and promotes autogenous shrinkage of the cementitious material [14,15]. In its turn, autogenous shrinkage causes cracking as deformation of the concrete element is restrained and shrinkage cannot occur freely. To limit autogenous shrinkage, again the addition of SAPs can provide the solution by using them as an internal curing agent. During mixing of the concrete material, the SAPs absorb part of the hydration water. Upon self-desiccation, this water is released and can be used for continuation of the hydration process. In this way, autogenous shrinkage can be reduced or even mitigated [16,17,18].

Although SAPs seem to be efficient in both mitigating autogenous shrinkage and promoting self-healing of concrete, the inclusion of these particles promote macro-pore formation upon release of the absorbed water. To improve the healing capacity of concrete, the amount as well as the size of the SAP particle should be relatively large (around 500 µm) in comparison to shrinkage reduction applications (around 100 µm) [19], which increases the pore volume of the cementitious mixture. As these voids are created within the hardened material, the properties of the matrix are affected too. Furthermore, due to internal curing, the cementitious matrix is also affected. The effect of SAP addition on mechanical properties is thoroughly studied and varies from small increases in strength [20], thanks to the continued hydration of cement, to considerable decreases [21,22] due to the large number of pores, depending on the water-to-binder ratio.

In order to counteract the possible decrease in mechanical performance, other additives can be used in combination with SAPs. In a previous study, the effect of nanosilica in mixtures with and without SAP was studied. As the noun implies, nanosilica is classified as a nanoreinforcement and can be considered as the smaller, engineered form of silica fume. Few studies have yet been performed on the addition of nanosilica to cementitious mixtures and results showed both an increase in mechanical properties and a lowering of the pore volume [23,24]. These beneficial effects are attributed to three different characteristics of the material. First of all, nanosilica acts like a filler of smaller pores thanks to the small particle size. Secondly, nanosilica is a pozzolanic material and is thus able to form hydration products at an older age. Moreover, the large surface area serves as nucleation site for the precipitation of Portlandite and accelerates the hydration progress.

In this research, SAPs, nanosilica and a combination of both materials are added to a mortar reference mixture in order to obtain identical mechanical properties compared to the reference and to promote the self-healing capacity of the mix. Preceding research showed that, using a specific amount and type of both superabsorbent polymer and nanoreinforcement, it was possible to decrease the risk of autogenous shrinkage cracking, while maintaining the compressive strength of the reference material [25]. As this mix design was reported to be effective for the reduction of autogenous shrinkage, the question arises as to whether the mixture also possesses the ability of healing its own cracks.

The paper investigates the influence of SAPs and nanosilica on the self-healing capacity of cementitious mortars. Prismatic specimens were cracked after 28 days of curing and their crack width was measured and fixed at around 150 µm. Over a period of 40 days, the specimens were subjected to wet–dry cycles to improve the self-healing ability. During this 40-day period, crack width measurements were performed at specific moments in time by means of an optical microscope. On top of this, a permeability set-up was used to measure the amount of water flowing through the crack mouth opening over a certain period of time. The combination of both techniques made it possible to conclude on the self-healing ability of the mixtures with SAPs and to compare the effects of different additives to the behaviour of the reference material.

## 2. Materials and Methods

### 2.1. Materials

In this research study, four mortar mixtures were made: a reference mixture, a mixture containing SAPs, a mixture with nanosilica and a mixture combining both nanosilica and SAPs. The reference mixture consisted of ordinary Portland cement CEM I 52.5N Strong (Holcim, Belgium), river sand (0/2), tap water and a superplasticizer, being MasterGlenium 51 (conc. 35%) from BASF, Belgium. The mix proportioning is as follows: binder: water: sand: superplasticizer = 1:0.35:2:0.004.

The superabsorbent polymer (SAP A) used is a copolymer of acrylamide and sodium acrylate with a particle size of 100±21.5 μm and is manufactured and provided by BASF, Germany. The amount of SAPs added to the mixture is fixed to 0.2% by mass of the cement. This value is slightly lower than the amount needed to completely mitigate autogenous shrinkage, being 0.24% by mass of the cement, as defined by Jensen et al. [17], to limit the strength loss due to macro-pore formation. To compensate for the water that the SAPs absorb, extra water is added to these mortar mixtures. The exact amount of water added is determined by a measurement of the flow, following NBN EN 1015-3 [26], which should equal the flow of the reference mixture. Concerning the nanoreinforcement, LUDOX^®^ HS-40 from W. R. Grace & Co.-Conn. (Antwerp, Belgium) is used. LUDOX^®^ is a colloidal dispersion, containing 40% by mass of synthetic amorphous silica in an aqueous medium. The density of this solution is equal to 1.3 g/cm^3^, whereas the nanosilica particles have a nominal diameter of 12 nm and a specific surface area between 198 and 258 m²/g. In the mixtures containing nanosilica, the silica replaces cement in an amount of 2% dry powder by mass of the binder, which is the amount needed for optimal strength increase [25]. Furthermore, an additional amount of superplasticizer is added to the mixtures with HS-40 to obtain the same flow compared to the reference mixture. The quantities of materials needed are summarized in Table 1. Note that the mass of HS-40 is the amount of colloidal solution used, being 40% nanosilica and 60% water. The amount of water present in the colloidal silica is then subtracted from the original amount of water, to obtain an equal water-to-cement ratio compared to the mixtures without nanosilica.

### 2.2. Sample Preparation and Crack Creation

The following procedure is based on the method used by Van Mullem et al. [27]. In order to perform the water permeability test, prismatic specimens were cast measuring 40 mm × 40 mm × 160 mm. To obtain a cast-in hole over the full length of the beam specimens, to allow for water flowing through the sample and leak out of the crack, a smooth steel bar of 5 mm in diameter was added inside the moulds at a height of 15 mm from the bottom of the specimen and centrally positioned with respect to the width of the sample. After a day of curing, the steel bar was removed from the moulds and the specimens were sealed with plastic foil until the day of cracking, which was 28 days. For each test series, meaning the different mixture compositions, six specimens were cast.

To evaluate the self-sealing and/or–healing efficiency of the different mixtures, a crack should be introduced. For this reason, a unidirectional carbon-fibre-reinforced polymer (CFRP) laminate, PC^®^ CARBOCOMP UNI (TRADECC, Belgium), was glued on top of each specimen by means of an epoxy resin two days before testing. After the total of 28 days of curing, the prisms were cracked by means of a three-point bending test with a Walter & Bai 15 kN testing machine (Löhningen, Switzerland). The laminate offered the possibility of introducing a single through-going crack, without full separation of the two beam halves. The crack width was, however, wider than the target crack width of 150 µm and needed, therefore, to be closed again. This was done by placing the specimens in a small metal framework and screwing the halves closer to each other. The crack width was then measured microscopically at the crack mouth opening (see Section 2.3) after each restraining trial until the target crack width was reached. This obtained crack width was maintained during the subsequent testing campaign.

**Figure 1:**
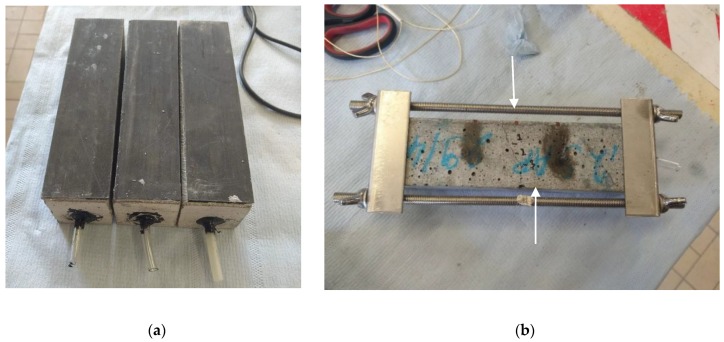
View of the prism samples: (**a**) top view with CFRP laminate and connected tubes and (**b**) bottom view with restraining framework showing a specimen with a crack in the middle part. The depicted arrows show the boundaries of the crack near the side.

Regarding the water permeability test, water should be able to flow through the crack mouth only. Therefore, one side of the hollow cores was sealed, while the other was equipped with a plastic tube to allow for connection to the permeability test set-up. The sides of the crack are sealed by means of an aluminium tape. The resulting samples can be seen in Figure 1, where both the specimens and the metal framework are shown.

After preparation of the samples, wet–dry cycles were performed during 40 days on five out of six samples: 23 h dry at 20 ± 2 °C and a relative humidity (RH) of 60% ± 5% and 1 h in water at 20 ± 2 °C per day. The remaining specimen was placed in an air-conditioned room at 20 ± 2 °C and 60% ± 5% RH. Measurement of the crack widths and water permeability were conducted at specific moments during this 40-day period and will be specified in the following sections.

### 2.3. Crack Width Measurements Using Microscopy

The crack width of each specimen was measured by means of an optical microscope (Leica S8 APO mounted with a DFC 295 camera) at the crack mouth. Three positions were chosen along the crack, where the opening would be measured. In each of these positions, a picture was taken using the microscope and per image, approximately 5 measurements of crack width opening were carried out at predetermined and fixed positions. The average of these 15 values is defined as the mean crack width.

The measurement of crack widths was performed at the start of the curing cycles and was repeated after 3, 7, 14 and 28 days of healing. The same positions as those chosen on the first day were then again photographed and the crack openings were measured at the same locations with respect to the first measurement to investigate the evolution of healing in time.

### 2.4. Permeability Test

**Figure 2:**
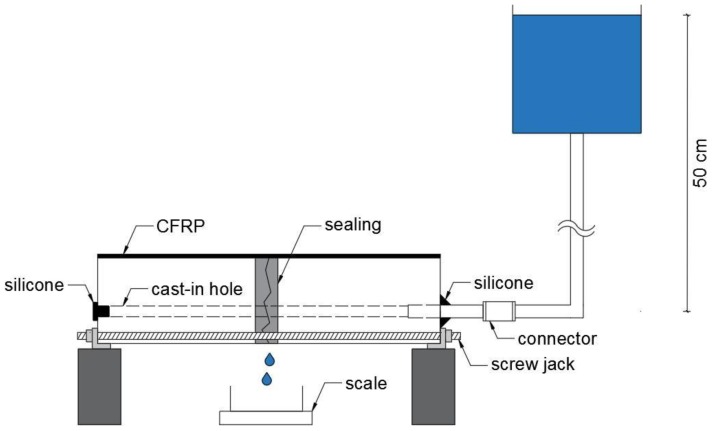
Set-up of the permeablity test (Van Mullem et al. [27]).

The set-up of the water permeability test is identical to the one proposed by Van Mullem et al. [27], also used in the FP7 project HEALCON [10], referred to as the ‘water flow setup’, and is depicted in Figure 2. A connection is made between the tube and a water reservoir holding a water head at a height of approximately 50 cm. The water head is kept constant during the test. The samples are placed above an automatic weighing scale, so that the water leaking out of the crack is continuously measured.

To avoid the presence of air inside the crack as much as possible, the samples were saturated prior to testing by placing them in water at 20 ± 2 °C for approximately 48 h. The test was conducted three times: right before the initiation of the wet–dry cycles, after 14 days and immediately after the 40-day healing period. This means that two interruptions of the wet–dry cycles were necessary, i.e., the one-hour dry period was skipped on day 13 and day 39 and the samples were tested 24 h later.

The mass of water leaking out of the crack was monitored for 10 min. The first minute was not included, to make sure no remaining air bubbles were influencing the flow of water, and a steady-state water flow was achieved. From the monitored mass of water, the average flow rate [g/min] was calculated.

### 2.5. Flexural and Compressive Strength

Next to the specimens made for evaluation of the self-healing capacity, a series of 12 prisms with identical dimensions of 40 mm × 40 mm × 160 mm were cast per mixture to obtain the flexural and compressive strength of the different mix designs. The specimens were kept in plastic foil at a temperature of 20 ± 2 °C and three samples per mixture were tested at an age of 3, 7, 28 and 90 days. To obtain the flexural strength, a three-point bending test was performed following ASTM C 348-14 [28], with a displacement rate of 0.5 mm/min. The broken halves were subsequently used for measurement of the compressive strength, conform ASTM C 349-18 [29], at a displacement rate of 1 mm/min.

## 3. Results and Discussion

### 3.1. Crack Width Measurements Using Optical Microscopy

**Figure 3:**
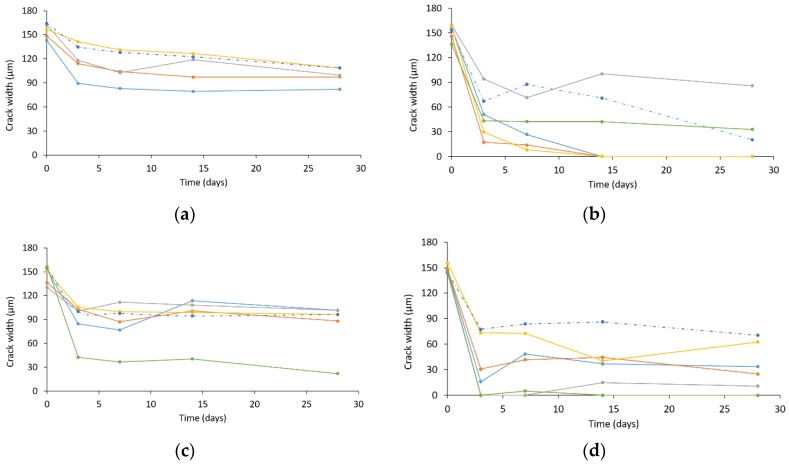
Evolution of crack width over time of (**a**) reference, (**b**) 0.2% SAP, (**c**) 2% HS-40 and (**d**) 0.2% SAP + 2% HS-40. The different curves represent the various specimens of each series that were tested.

As explained in Section 2.3, the initial crack widths were measured at approximately 15 different locations along the crack mouth. Afterwards, at 3, 7, 14 and 28 days of curing in wet–dry conditions, the crack width was measured at these same locations to evaluate crack closure. The evolution of the average crack widths is shown in Figure 3 for the four different testing series. It should be noted that in the case of the reference series, only five samples could be monitored as dirt was trapped inside the crack of the last sample, which made accurate measurements impossible. The results of the sample that was not subjected to wet–dry cycles is shown by means of a dotted line.

It can be seen from Figure 3a that the average initial crack width of all reference samples was close to 150 µm. Over time, the cracks close partly, which means limited crack healing. The largest decrease in crack opening happens during the initial days, which is mostly the result of a high amount of unhydrated cement particles present in the mixture. Afterwards, the crack closure slows down towards a more constant value and this is not below 80 µm. Rather unexpected is the slight increase in the average crack width of one of the samples between 7 and 14 days. This is due to debris detaching from the crack walls at one of the measuring locations: the crack width then increases locally and the average crack width follows this trend. Regarding the sample kept in dry conditions, an identical trend can be noticed compared to the other reference samples: the largest extent of crack closure occurs within the initial days. Moreover, no difference in the final crack width can be seen when comparing this sample to the specimens cured in wet–dry cycles.

A strong resemblance in crack closure can be observed between the reference series and the one containing 2% of nanosilica, depicted in Figure 3c. As stated above, the decrease in crack width is the highest within the first days after cracking due to the availability of unhydrated cement, while the values tend to attain a constant value subsequently. In terms of absolute value, the sample series containing nanosilica has one outlier, which shows a much higher decrease in crack opening. This resulted from the fact that the crack was fully closed after 3 days in one of the three measuring positions chosen, drastically lowering the average crack width.

In the case of SAP addition to the reference mixture (Figure 3b), the average initial crack widths are within the same range as for all other samples. The cracks close more quickly within the first days of application of the wet–dry cycles and further change later on. The decrease in crack width is more significant in the case of SAP addition and, for three out of five samples, the crack width reaches zero after 14 days of wet–dry curing. Similarly to the reference series, one of the samples shows a significant increase in average crack width between 7 and 14 days, which was due to the enlarged opening in one of the three picture positions. The remaining average crack width opening of this same specimen also remains significantly larger than the one of all other samples containing SAPs. A possibility is the washing out of SAP particles, due to a locally wider crack opening. This assumption will be further substantiated in Section 3.2. Concerning the sample held in dry conditions, the decrease in average crack width was lower within the first days, as no water was available for sample healing. However, after four weeks, significant crack closure was seen within this specimen, mostly due to the use of ambient moisture that is captured and released by the SAPs. This effect was also reported in the literature [8].

Crack width measurements of the combination of both SAPs and HS-40 can be seen in Figure 3d. The resulting effect of both materials on the crack opening can be interpreted as a combination of the action of the SAPs and the nanosilica: the SAPs account for a better closure of cracks compared to the simple nanosilica-containing specimens, wherethe nanosilica densifies the matrix. This matrix densification limits the access of water to unhydrated cement particles and causes a lower degree of crack closure with respect to the SAP series.

**Figure 4:**
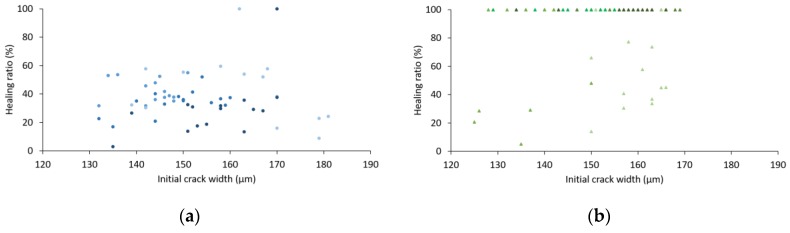

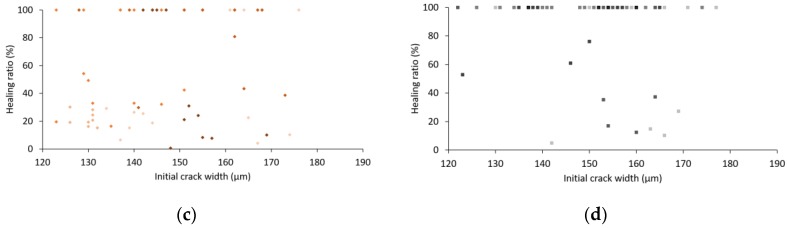
Healing ratio in % versus initial crack width of (**a**) reference, (**b**) 0.2% SAP, (**c**) 2% HS-40 and (**d**) 0.2% SAP + 2% HS-40.

By means of the crack width measurements, the crack healing efficiency could be evaluated. The so-called healing ratio was calculated by:

$$Healing\;ratio\left(\%\right)=\frac{w_{i}-w_{f}}{w_{i}}\times 100$$

with *w_i_* the initial crack width [µm] and *w_f_* the final crack width (µm), here measured after 28 days of wet–dry cycles. The result can be seen in Figure 4 plotted against the initial crack widths. Note that all measuring positions of samples held in wet–dry cycles are included in these graphs. The different colour shades represent the different samples in one series.

Figure 4a shows the healing ratio of the reference samples and it is noticed that the initial crack width (in the range of 120 µm to 190 µm) does not seem to have any influence on the healing ratio. Also, results pointed out that complete healing was not feasible in the reference samples, as only two specific locations out of 60 showed a closed crack after 28 days of wet–dry cycles.

The healing ratio of nanosilica specimens shows a high variability on the results: whereas some measuring positions were found to be 100% closed, others only showed a decrease in crack width of 20%–40%.

**Figure 5:**
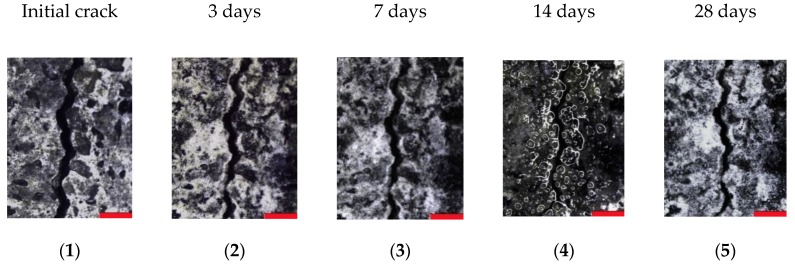
Evolution of crack closure as seen in microscopic images of a reference sample at (**1**) initial measurement and after (**2**) 3 days, (**3**) 7 days, (**4**) 14 days and (**5**) 28 days of wet-dry cycles. The scale bar at the bottom right of every micrograph represents 500 µm.

**Figure 6:**
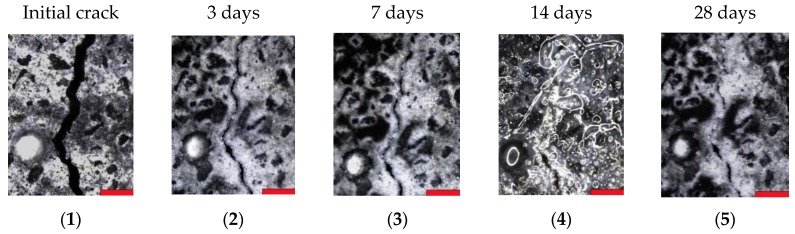
Evolution of crack closure as seen in microscopic images of a SAP sample at (**1**) initial measurement and after (**2**) 3 days, (**3**) 7 days, (**4**) 14 days and (**5**) 28 days of wet-dry cycles. Clear crystals are formed in the SAP-containing specimen. The scale bar at the bottom right of every micrograph represents 500 µm.

In comparison to their respective reference series, the samples containing SAPs showed a significant amount of closed crack positions after wet–dry curing, which is shown in Figure 4b,d. The closure of cracks is again independent of the initial crack width and is due to the deposition of healing products inside the crack. This is confirmed by the microscopic analysis. Examples of one reference sample and one specimen containing SAPs are depicted in Figure 5 and Figure 6 respectively, showing five pictures of the different measuring moments. The red bars in every figure have a width of 500 µm. The pictures taken at 14 days of healing show a lot of reflection and bubbles present on the specimen’s surface, this is because of the fact that samples were saturated beforehand to conduct the water permeability test and were still humid upon measuring the crack widths.

It can be seen that the crack inside the reference sample shows partial healing over time, whereas for the SAP specimen, the amount of healing product is significantly increased compared to the reference. After 28 days, the considered crack position of the SAP specimen is almost fully closed by the deposition of a whitish crystal, predominantly calcium carbonate in combination with further hydration products [9], compared to the narrow opening seen for the reference sample.

### 3.2. Permeability Test

A permeability test was conducted at three moments in time on the samples subjected to wet–dry cycles. As the samples needed to be saturated before testing, to limit the amount of air present inside the cracks, the test was performed before and after the period of 40 wet–dry cycles and only once in between those cycles, after 14 days of wet–dry curing.

**Figure 7:**
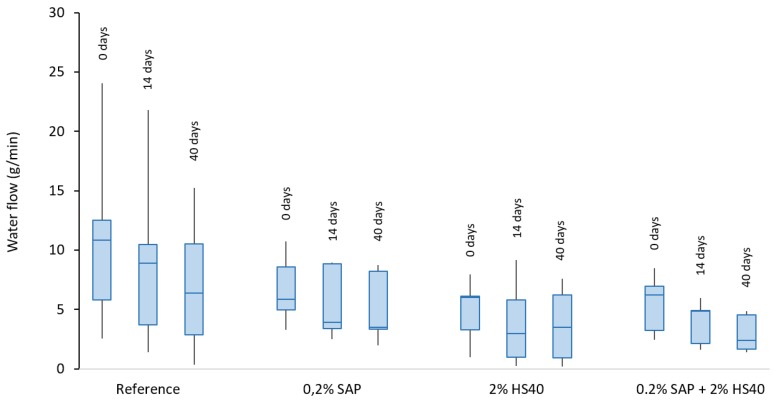
Average water flow of all series measured at 0, 14 and 40 days of wet–dry cycles. The boxes show the first, second and third quartile, being the lower, middle and upper line, respectively. The whiskers determine the minimum and maximum values obtained.

The amount of water leaking out of the crack opening was continuously measured over a time period of 5–10 min, depending on the time needed to reach a steady flow. The water flow was then calculated as the amount of water per minute that leaks out. The results are summarized by means of boxplots in Figure 7. for the three measuring moments. The boxes are delimited by means of the first and third quartile and show the median value in between. The minimum and maximum flow obtained per series are indicated by means of the whiskers.

It is noticed that the flow values obtained for the reference series are widely spread, despite the similar crack widths induced, and at all three measuring occasions. Over time, as the samples are healed, the water flow decreases. After 40 days of wet–dry cycles, one of the samples was shown to have an almost totally sealed crack, whereas the others still allowed for a significant amount of water leaking out.

The water flow through the SAP specimens showed an immediate sealing effect due to the SAP action compared to the reference samples for the initial measurement, as can be seen from the decreased flow values obtained. Upon contact with water, the SAP particles inside the crack swell and block the water flow. Although the SAPs are holding the water, stopping it from leaking out of the crack, it can be seen that perfect sealing was not obtained due to the small amount of SAP particles present in the mixture. When the samples were placed in wet–dry conditions, healing of the crack openings takes place and the permeability decreases even more. However, between 14 and 40 days, the healing effect does not seem to improve significantly. On the one hand, this result can be explained by the fact that most of the unhydrated cement particles have already reacted upon contact with water and no further hydration occurs. On the other hand, there is the possibility that, in some of the samples, SAP particles might have been washed out of the crack. Although this assumption is not very likely, as the SAPs will swell up to a particle size of around 250 µm and become larger than the initial crack width, the hypothesis holds in the case that an enlargement of the crack width opening occurs in some of the samples. Furthermore, to have complete sealing of a crack, higher amounts of SAPs are needed. The amount in this research, which is 0.2% of the weight of cement, is typically used to mitigate autogenous shrinkage, whereas amounts of up to 1% of cement weight are used for sealing and healing purposes. For sealing, this high amount is needed to completely physically block a crack from intruding fluids [30,31]. Using a lower amount of SAPs results in a partial, but still noteworthy, sealing effect. 

Evolution in permeability results in HS-40 specimens is closely related to the reference series, notwithstanding the considerably lowered water flow values obtained in case of nanosilica addition. Identical to the healing of the other mix series, the water flow decreases over time, except for one sample showing a slightly lowered water flow at 14 days compared to the value at 40 days, which greatly influences the median value at 14 days.

Comparison of SAP/HS-40 addition to both individual SAP and HS-40 mixes shows that no noteworthy difference can be noticed compared to the water flow values of the SAP and HS-40 series. However, the action of the SAPs can be seen in the healing during wet–dry cycles, as the extent of crack healing is more pronounced in the case of SAP + HS-40 compared to HS-40 samples.

### 3.3. Flexural and Compressive Strength

**Figure 8:**
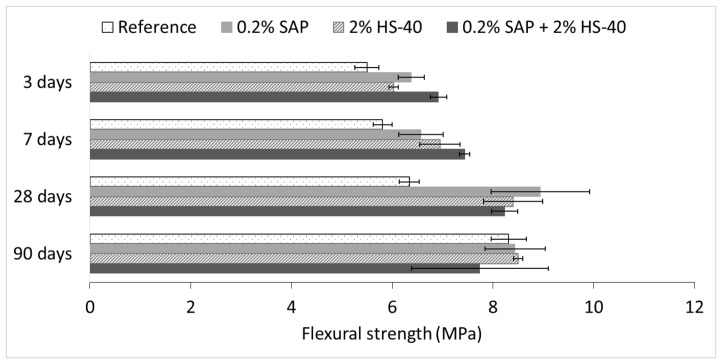
Flexural strength of all series measured at 3, 7, 28 and 90 days.

**Figure 9:**
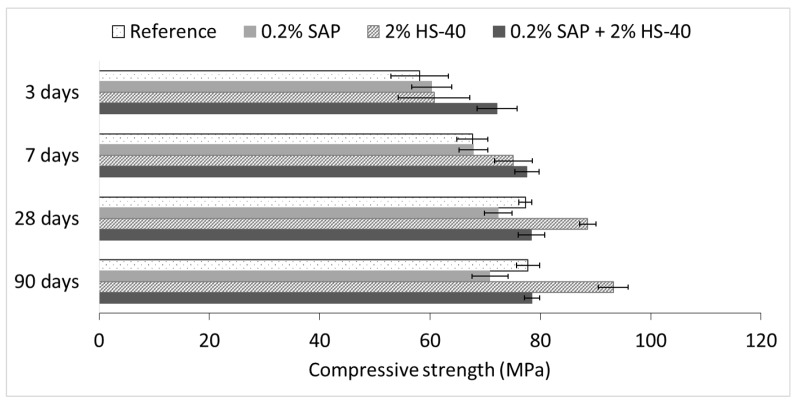
Compressive strength of all series measured at 3, 7, 28 and 90 days.

As explained in the introduction, the addition of superabsorbent polymers promotes the formation of macro-pores, which can strongly affect the mechanical properties of the cementitious material. To counteract the possible decrease in strength, nanosilica was added as a reinforcing material. The effect of both materials on the flexural and compressive strength was, therefore, evaluated experimentally at different curing ages. The results of both tests are shown in Figure 8 and Figure 9 for flexural and compressive strength, respectively.

As can be seen in Figure 8 the flexural strength increases over time for all series tested. Both SAP and nanosilica have a noticeable influence on the flexural strength. Within the first 28 days, all mixtures containing additives show a higher strength during the three-point bending test as compared to the reference series. For SAP addition, this can be explained by the fact that the water absorbed by the SAPs is released and can be used for further hydration, while the influence on the flexural strength depends strongly on the size of voids formed. Also, the execution of the three-point bending test leads to a preferential pathway for crack formation. As different amounts of voids can be present on the crack path for different samples, the standard deviation on the results is increased. In the case of nanosilica addition, the nanosilica acts as the nucleation site, increases the rate of C–S–H production and contributes to strength development. After 90 days of curing, the flexural strength of all mixtures is approximately equal.

Regarding the compressive strength, summarized in Figure 9 an identical increasing trend over time can be seen as compared to the flexural strength. However, in this matter, the addition of SAPs lowers the strength values by creation of macro-pores. Identical to the increased flexural strength, the addition of nanosilica has a significant positive effect on the compressive strength. At 28 days, the maximum strength values of nanosilica-containing mixtures are about 14% higher than the ones obtained for the reference samples. The inclusion of both SAP and nanosilica combines the effects seen in the separate mixes and provides a compressive strength that is comparable to the strength of the reference series from 28 days onwards. In this way, the negative influence of the macro-pore formation is counteracted, yielding a material with the same strength properties as the reference material but with superior sealing and healing characteristics.

## 4. Conclusions

This research studied the self-healing potential of mortar mixtures containing superabsorbent polymers and nanosilica. To fully understand the action of both additives, four different mortar mixtures were made, and there were the following: reference, 0.2% SAP, 2% HS-40 and a combination of both SAP and nanosilica. The self-healing efficiency of these mixes, under the application of wet–dry cycles, was evaluated by means of microscopy measurements of the crack width and a water permeability experiment, whereas the mechanical properties were assessed by means of three-point bending and compressive strength tests.

Whereas the SAP-containing mixtures were initially designed to mitigate autogenous shrinkage, an increase in the crack closing capability was noticed. Cracks with an initial width of around 150 µm were shown to have healed over time upon application of wet–dry cycles for all four mix series. However, whereas the closure of reference and nanosilica samples was limited to 60%, samples containing SAP and both SAP and nanosilica reached 100% crack closure in more than 75% of the measurement positions chosen. This was concluded from crack width measurements in microscopy pictures taken at different curing ages.

Additionally, water permeability tests showed the immediate sealing effect of SAP samples compared to the reference series. Also, the amount of water leaking out of the cracks decreased with curing time, confirming the continued healing of cracks in all mixture series.

Finally, it was seen that the addition of superabsorbent polymers lowered the compressive strength of the mortar mixtures, due to macro-pore formation. On the contrary, nanosilica increases the mechanical properties thanks to the nucleation effect and its pozzolanic behaviour. By using both additives together, the mechanical performance was not affected in comparison to the reference mix. This opens the pathway to use this material in building applications. Whereas for self-healing purposes, an amount of SAP higher than 1% by mass of the binder is reported to be necessary [7], it was found that the mixtures under study, which were initially designed to mitigate autogenous shrinkage, showed superior sealing and healing characteristics compared to the reference material, while maintaining the mechanical properties.

## Figures and Tables

**Table 1:** Mix proportions in kg/m^3^.

Mix Series	Cement	Water	Sand	Superplasticizer	SAP	HS-40
Reference	580	203	1160	2.32	-	-
0.2% SAP	580	233.16	1160	2.32	1.16	-
2% HS-40	568.4	185.6	1160	4.41	-	29
0.2% SAP + 2% HS-40	568.4	215.76	1160	4.41	1.16	29

## References

[1] Cailleux E., Pollet V. Investigations on the development of self-healing properties in protective coatings for concrete and repair mortars. Proceedings of the 2nd International Conference on Self-Healing Materials.

[2] Van Tittelboom K., de Belie N. (2013). Self-Healing in Cementitious Materials-A Review. Materials.

[3] Van Tittelboom K., Wang J., Araujo M., Snoeck D., Gruyaert E., Debbaut B., Derluyn H., Cnudde V., Tsangouri E., van Hemelrijck D. (2016). Comparison of different approaches for self-healing concrete in a large-scale lab test. Constr. Build. Mater..

[4] Huang H., Ye G., Qian C., Schlangen E. (2016). Self-healing in cementitious materials: Materials, methods and service conditions. Mater. Des..

[5] Kim J., Schlangen E. Super absorbent polymers to stimulate self healing in ECC. Proceedings of the 2nd International Symposium on Service Life Design for Infrastructures (SLD2010).

[6] Snoeck D., van Tittelboom K., de Belie N., Steuperaert S., Dubruel P. The use of superabsorbent polymers as a crack sealing and crack healing mechanism in cementitious materials. Proceedings of the 3rd International conference on concrete repair, rehabilitation and retrofitting.

[7] Snoeck D., van Tittelboom K., Steuperaert S., Dubruel P., de Belie N. (2014). Self-healing cementitious materials by the combination of microfibres and superabsorbent polymers. J. Intell. Mater. Syst. Struct..

[8] Snoeck D., de Belie N. (2015). Repeated autogenous healing in strain-hardening cementitious composites by using superabsorbent polymers. J. Mater. Civ. Eng..

[9] Snoeck D., de Belie N. (2019). Autogenous healing in strain-hardening cementitious materials with and without superabsorbent polymers: An 8-year study. Front. Mater..

[10] Gruyaert E., Debbaut B., Snoeck D., Diaz P., Arizo A., Tziviloglou E., Schlangen E., de Belie N. (2016). Self-healing mortar with pH-sensitive superabsorbent polymers: Testing of the sealing efficiency by water flow tests. Smart Mater. Struct..

[11] Lee H., Wong H., Buenfeld N. Self-sealing cement-based materials using superabsorbent polymers. Proceedings of the International RILEM Conference on Use of Superabsorbent Polymers and Other New Additives in Concrete.

[12] Powers T., Brownyard T. (1946). Studies of the physical properties of hardened Portland cement paste. J. Proc..

[13] Hansen T. (1986). Physical structure of hardened cement paste. A classical approach. Mater. Struct..

[14] Jensen O., Hansen P. (1996). Autogenous deformation and change of the relative humidity in silica fume-modified cement paste. Mater. J..

[15] Lura P., Jensen M., van Breugel K. (2003). Autogenous shrinkage in high-performance cement paste: An evaluation of basic mechanisms. Cem. Concr. Res..

[16] Mechtcherine V. (2016). Use of superabsorbent polymers (SAP) as concrete additive. RILEM Tech. Lett..

[17] Jensen O., Hansen P. (2001). Water-entrained cement-based materials: I. Principles and theoretical background. Cem. Concr. Res..

[18] Snoeck D., Jensen O., de Belie N. (2015). The influence of superabsorbent polymers on the autogenous shrinkage properties of cement pastes with supplementary cementitious materials. Cem. Concr. Res..

[19] Van Tittelboom K., Snoeck D., Wang J., de Belie N. Most recent advances in the field of self-healing cementitious materials. Proceedings of the 4th International conference on Self-Healing Materals (ICSHM 2013).

[20] Hasholt M., Jespersen M., Jensen O. Mechanical properties of concrete with SAP part I: Development of compressive strength. Proceedings of the International RILEM Conference on Use of Superabsorbent Polymers and Other New Additives in Concrete.

[21] Craeye B., Geirnaert M., de Schutter M. (2011). Super absorbing polymers as an internal curing agent for mitigation of early-age cracking of high-performance concrete bridge decks. Constr. Build. Mater..

[22] Mignon A., Snoeck D., D’Halluin K., Balcaen L., Vanhaecke F., Dubruel P., van Vlierberghe S., de Belie N. (2016). Alginate biopolymers: Counteracting the impact of superabsorbent polymers on mortar strength. Constr. Build. Mater..

[23] Du H., Du S., Liu X. (2014). Durability performances of concrete with nano-silica. Constr. Build. Mater..

[24] Said A., Zeidan M., Bassuoni M., Tian Y. (2012). Properties of concrete incorporating nano-silica. Constr. Build. Mater..

[25] Lefever G., Tsangouri E., Snoeck D., Aggelis D., de Belie N., van Vlierberghe S., van Hemelrijck D. (2019). Combined use of superabsorbent polymers and nanosilica for reduction of restrained shrinkage and strengt compensation in cementitious mortars. Constr. Build. Mater..

[26] Belgisch Instituut Voor Normalisatie (BIN) (1999). Methods of Test for Mortar Masonry-Part 3: Determination of Consistence of Fresh Mortar (by Flow Table).

[27] Van Mullem T., Gruyaert E., Debbaut B., Caspeele R., de Belie N. (2019). Novel active crack width control technique to reduce the variation on water permeability results for self-healing concrete. Constr. Build. Mater..

[28] ASTM International (2018). ASTM Standard C 348-18: Standard Test Method for Flexural Strength of Hydraulic-Cement Mortars.

[29] ASTM International (2018). ASTM Standard C 349-18: Standard Test Method for Compressive Strength of Hydraulic-Cement Mortars (Using Portions of Prisms Broken in Flexure).

[30] Snoeck D., Steuperaert S., van Tittelboom K., Dubruel P., de Belie N. (2012). Visualization of water penetration in cementitious materials with superabsorbent polymers by means of neutron radiography. Cem. Concr. Res..

[31] Snoeck D., van den Heede P., van Mullem T., de Belie N. (2018). Water penetration through cracks in self-healing cementitious materials with superabsorbent polymers studied by neutron radiography. Cem. Concr. Res..

